# A Case of Chronic Pachymeningitis Following the Course of the Superior Longitudinal Sinus, and Apparently the Result of Chronic Syphilitic Rhinitis

**Published:** 1899-07

**Authors:** Eugene R. Corson

**Affiliations:** Savannah, Ga.


### A CASE OF CHRONIC PACHYMENINGITIS FOLLOWING THE COURSE OF THE SUPERIOR LONGITUDINAL SINUS, AND APPARENTLY THE RESULT OF CHRONIC SYPHILITIC RHINITIS.

BY EUGENE R. CORSON, B.S., M.D.

SAVANNAH, GA.

The following case seems of sufficient interest in its pathological bearings to be reported. If correct in my interpretation, it must be of some rarity, for I find in our literature scant mention of any relationship between brain and nose disease.

J. B., pilot, aged 59, contracted syphilis twenty years ago, for which he was treated in a desultory way. He was at times a hard drinker, which added to the severity of the syphilitic troubles. The tertiary lessons were pronounced, especially the rheumatism, the tibial nodes, and a rhinitis from which he never was entirely free. Several pieces of bone were discharged from the nose; one piece in my possession is a large scale from the vomer. There was for many years a constant foul discharge; there was complete anosmia. In spite of this taint and his periodical drinking and otherwise irregular living, he kept fairly robust, and was able to do his work as a pilot to the satisfaction of his associates.

Three years ago he was taken suddenly with convulsions, epileptic in their character, and following each other in rapid succession; in other words, a typical *status epilepticus*. On recovering he was left with a paresis of the right arm and leg, and it soon became apparent that his mind was weakened. His memory was so poor that he was compelled to give up his work. This loss of memory was limited largely to recent events, the recollection of his youth and early manhood were still good, at times remarkably so. He complained often of severe headaches, frontal and bregmatic. There is no history of epistaxis, despite the rhinitis and the evident congestion of the brain.

One year after this attack he had another similar one, and I saw him at the time, a genuine *status epilepticus* from which, after about thirty convulsions, he recovered without any increase in the paresis. The mind, however, was weaker. The urine showed a trace of albumen.

Several days preceding the final attack in which he died there was trembling and titubation in the leg; at times it would be suddenly drawn up, showing cortical irritation. Convulsions started suddenly after a hearty meal which he seemed to enjoy. He had about twenty convulsions before his death.

I had permission to remove the brain only, and the post-mortem had to be made on time in a small room with a poor light and unassisted. In using the saw the dura and brain were slightly injured, and there was an escape of about a half pint of serum. The dura over the hemispheres was removed with the brain which was carefully bisected and placed immediately in a 5 per cent. solution of formalin, and the parts were not examined until the tissues had been hardened.

The hemispheres gave evidence of a marked passive hyperemia, all the vessels of the pia being distended. The lateral ventricles and the third ventricle with the foramen of Munroe were perceptibly enlarged. There was no evidence whatsoever of the middle commissure, or *commissura mollis*, the walls of the third ventricle showing the smooth ependymal lining. I am aware that this delicate commissure is often ruptured in removing the brain and thus may lead one to think it absent, but in this case there would be some evidence of the rupture; here there was no sign of it. The basilar artery and the circle of Willis and its immediate branches showed atheromatous patches. After careful search I could find no evidence of rupture at any time, and apoplexy could be eliminated. On removing the dura it was found adherent to the pia the whole length of the superior longitudinal sinus, a streak of inflammation one and a half centimeters wide. There was some adhesion on the right side following the sinus, but the evidence of pachy- and leptomengitis on this side were much less evident, and the dura could be detached without much difficulty.

The walls of the longitudinal sinus were much thickened and the sinus itself so constricted that only the thinnest stream could trickle through. This constriction extended up to about four centimeters of the torcular herophili. There were no clots in any of the sinuses. The evidences of meningitis were especially noticeable over the left leg and arm centers, which explained the right-sided paresis of arm and leg. The inferior longitudinal sinus or vein was completely obliterated up to within two centimeters of the straight sinus.

The veins of Galen seemed to me somewhat enlarged, at once suggesting that perhaps they had acted as collaterals for the constricted longitudinal sinus.

To sum up my findings: Here was a brain showing a pachymeningitis limited largely to the course of the superior longitudinal sinus, with a leptomeningitis, with some dilatation of the lateral and third ventricles, with an obliterated inferior longitudinal sinus, with a history of syphilis and a chronic syphilitic rhinitis, especially severe and of long standing. With this before us it seems reasonable to infer that the brain findings are but the result of the nose trouble through the close connection between the two by the ethmoid and the foramen cecum, the latter giving a direct venous connection. This seems to me all the more probable because the meningitis, though of a chronic character, was apparently entirely limited to the course of the longitudinal sinus, the direct continuation of the veins in the nose.

I admit that the chain of evidence as to this relationship is far from complete, because I failed to examine the ethmoid and the foramen cecum with its vein. It seems to me, however, the only view which can harmonize the clinical history with the post-mortem findings.

The relationship of middle ear and mastoid disease with brain trouble is of daily observation. The relationship of disease within the orbit with brain trouble through the orbital veins and the cavernous sinus has been not infrequently noted, but it seems that brain trouble as a result of nose trouble must be a rare condition. And the reason is quite evident. In mastoid disease there is no easy outlet for pus; this holds less with orbital trouble; in the nose the drainage is free enough. In the literature at my disposal I can find nothing comparable to my own case. It must be rare. The subject is of sufficient importance to warrant a report of the case if for nothing else than to call forth a discussion.

